# What’s distressing about having type 1 diabetes? A qualitative study of young adults’ perspectives

**DOI:** 10.1186/1472-6823-13-25

**Published:** 2013-07-25

**Authors:** Myles Balfe, Frank Doyle, Diarmuid Smith, Seamus Sreenan, Ruairi Brugha, David Hevey, Ronan Conroy

**Affiliations:** 1 Department of Public Health Medicine and Epidemiology, Royal College of Surgeons in Ireland, St. Stephen’s Green, Dublin, Ireland; 2 Department of Sociology, University College Cork, Cork, Ireland; 3 Department of Psychology, Royal College of Surgeons in Ireland, St. Stephen’s Green, Dublin, Ireland; 4 Endocrinology Department, Beaumont Hospital, Dublin, Ireland; 5 Endocrinology Department, Connolly Hospital, Dublin, Ireland; 6 Department of Psychology, Trinity College Dublin, Dublin, Ireland

**Keywords:** Young adult, Distress, Qualitative, Type 1 diabetes, Emerging adulthood

## Abstract

**Background:**

Diabetes distress is a general term that refers to the emotional burdens, anxieties, frustrations, stressors and worries that stem from managing a severe, complex condition like Type 1 diabetes. To date there has been limited research on diabetes-related distress in younger people with Type 1 diabetes. This qualitative study aimed to identify causes of diabetes distress in a sample of young adults with Type 1 diabetes.

**Methods:**

Semi-structured interviews with 35 individuals with Type 1 diabetes (23–30 years of age).

**Results:**

This study found diabetes related-distress to be common in a sample of young adults with Type 1 diabetes in the second phase of young adulthood (23–30 years of age). Diabetes distress was triggered by multiple factors, the most common of which were: self-consciousness/stigma, day-to-day diabetes management difficulties, having to fight the healthcare system, concerns about the future and apprehension about pregnancy. A number of factors appeared to moderate distress in this group, including having opportunities to talk to healthcare professionals, attending diabetes education programmes and joining peer support groups. Young adults felt that having opportunities to talk to healthcare professionals about diabetes distress should be a component of standard diabetes care.

**Conclusions:**

Some aspects of living with diabetes frequently distress young adults with Type 1 diabetes who are in their twenties. Clinicians should facilitate young adults’ attendance at diabetes education programmes, provide them with opportunities to talk about their diabetes-related frustrations and difficulties and, where possible, assist in the development of peer-support networks for young adults with diabetes.

## Background

Diabetes is associated with psychological morbidity [1]. Anxiety and depression are common in individuals with diabetes, both with Type 1 and Type 2 [1-10], with some studies finding that diabetes significantly increases the odds of comorbid depression [10,11]. Psychological morbidity is clinically important for people with diabetes because it is often associated with diabetes management problems [1,4].

Most research into psychological morbidity in individuals with Type 1 diabetes has focused on serious psychopathology. Researchers have recently begun to argue, however, that a much larger number of individuals with diabetes experience sub-clinical diabetes-related distress than experience above-threshold psychological disorders, and that this distress may impact glycaemic control more than clinical disorders [2,11]. Diabetes distress is a general term that refers to the emotional burdens, stressors and frustrations that stem from managing diabetes, a severe, complex condition [12,13]. Recent research on Type 2 diabetes has found that distress begins to influence diabetes-specific behavioural indicators at much lower levels than was previously considered [12]. Although diabetes distress has traditionally been seen as a symptom of clinical depression, researchers are now beginning to argue that depression should be seen as one form of diabetes distress [2,14]. Qualitative studies suggest that many people who consider themselves to be distressed by their diabetes do not necessarily consider themselves to be depressed by it [11,14]. To date, distress in people with diabetes (Type 1 and 2) has usually been measured and studied quantitatively [2,11,15,16].

Depression and distress are both prevalent in individuals with Type 1 diabetes [17-22]. Little research has been conducted specifically on distress in young adults (individuals between 18 and 30 years of age) with diabetes [4], though distress is also considered to be prevalent in this age group [23]. For example 59% of young adults in Scott et al.’s study [24] said that stress management was the second most important issue that they wanted to talk to healthcare professionals about. Hislop et al. [4] used the CES-D depression scale to examine depression prevalence in a sample of 92 young adults with diabetes and found 35% reported depressive symptoms. These authors argued that the depression scale that they used in the study was likely identifying symptoms of diabetes distress.

The majority of empirical and conceptual research on distress in young adults with diabetes has focused on young adults in their late teens and early twenties. Distress arises at this time of life from the strains and difficulties that young adults face balancing their diabetes management activities with their need to engage in normal developmental activities [25,26].

Drawing on the work of Arnett [27] diabetes researchers argue that young adulthood is a divided developmental stage, composed of an early transitional phase (approximately 18–22 years of age) but also a later one (approximately 23–30 years of age) that is characterized by increased lifestyle stability [3,25,28-30]. Young adults in their twenties are more future conscious and concerned about their diabetes management than their younger counterparts [3]. They tend to lose their adolescent sense of invulnerability [31].

Young adults in this range also experience distress, and some experience increased distress as they transition through their twenties [4,7,32-34]. Why these older individuals experience diabetes distress is an issue that has not receive significant attention from researchers, though existing research provides some indications. One reason may be that the mid to late twenties may be just as unstable a life period for some young people as the late teens and early twenties. Arnett [35] notes that residential mobility is actually at its maximum for young people in their mid-20s, and that the 20s can be years of frequent changes in occupation, educational status and personal relationships [35]. Young adults in this age range are transitioning to parenthood, to long-term personal relationships, to careers, to general adult health services, all the while trying to effectively manage their diabetes. These competing health and developmental priorities can lead some young people to feel overwhelmed [1]. While clinicians have put substantial efforts into helping younger adults with Type 1 diabetes manage distress, for example by developing specialist young adult services, distress in young adults in their mid to late 20s has not received the same attention. Young people in this age are generally treated in adult services where psychological support is lacking [4].

This qualitative study investigates factors that cause diabetes distress in young adults with Type 1 diabetes aged 23–30 (the age range that diabetes researchers refer to as the ‘second phase of emerging adulthood’) [1,3]. Understanding the factors that drive distress is crucial for constructing appropriate interventions to combat it [12].

## Methods

This study used a qualitative methodology and was conducted in Ireland. It received ethical approval from the research ethics committee of the Royal College of Surgeons in Ireland.

The study used a purposeful sampling method, the most common type of qualitative sampling technique [36]. The aim of this method was to recruit a sample of approximately 30 young adults with Type 1 diabetes, 23–30 years of age.

Young adults (n = 32) were recruited primarily from an Irish Facebook support group for young adults with Type 1 diabetes, and from the Facebook page of Diabetes Ireland, Ireland’s leading diabetes charity. Recruitment advertisements indicated that the project was looking to talk to young adults with Type 1 diabetes who were between 23 and 30 years of age. After the interviews with Facebook respondents were completed an additional three young adults were recruited from a specialist young adult clinic in Ireland. The themes that arose in the interviews with the three young adults from the hospital setting were similar to those in the interviews with the young adults who were recruited from Facebook and therefore the researchers did not seek to recruit more participants. Twenty-nine women and six men responded to the study. Since the purpose of the study was to determine factors that cause distress in young adults in general (and *not* to differentiate between the accounts of male and female interviewees), and because similar themes arose in the six male and twenty-nine female interviews, male and female interviewees’ results are reported together. Previous studies that have used qualitative methods to investigate diabetes distress have also used unequal number of men and women [37,38]. See Table 1 for young adults’ demographic characteristics.

**Table 1:** Demographic characteristics of young adults who took part

	**N = 35**
Age (mean, SD)	26.9 (2.67)
Female	82.9%
Numbers of years has diabetes (mean, SD)	11.5 (5.6)
A1c (mean, SD)	7.95 (.76)/63 mmol
Number of blood tests per day (mean, SD)	4.47 (1.98)
On pump	26.5%
Educated to degree level	93.9%
Private health insurance	64% (only 28 interviewees answered this question)

The overall sample size (35 young adults) is within best practice guidelines for studies based on semi-structured qualitative interviews [39,40]. Table 2 indicates that the study reached data saturation at interview 3; every interview beyond that served to confirm the themes that emerged in the first three interviews, rather than introduce new themes.

**Table 2:** Range of distress-related themes in each interview

**Interviewee**	**Struggles with health system**	**Pregnancy**	**Day-to-day management problems**	**Type 2 diabetes**	**Self-consciousness/stigma**	**Complications/Future**	**Media Representations of Type 1 diabetes**	**Limiting/gets in the way of things**
1	X	X		X		X	X	X
2	X	X				X		X
3		X	X	X	X	X	X	
4	X	X	X					X
5			X		X			X
6	X	X	X		X	X		X
7	X	X	X	X	X	X		X
8	X			X		X	X	
9		X				X		X
10					X	X		X
11	X			X				X
12	X		X		X	X		X
13			X	X	X			X
14	X		X		X	X		X
15								
16			X	X		X		
17			X			X		
18	X							
19	X	X	X	X		X		X
20	X			X	X	X		X
21	X			X	X	X		
22	X	X	X	X		X		X
23								
24			X					X
25	X		X					X
26								
27	X		X					X
28		X	X	X	X	X		X
29					X			X
30			X	X		X	X	
31				X		X		
32		X	X			X		X
33		X		X		X		
34						X		
35								

X = this theme was present in this interviewees’ account.

All interviews were conducted by the first author who at the time that the study was conducted was thirty-one years of age. Seventeen interviews were conducted over the telephone and eighteen in person. Interviews lasted 34–86 minutes, with telephone interviews being shorter. The development of the interview schedules was informed by literature review and expert opinion (feedback from the third author). The interview schedule was piloted with two people with diabetes who were in their early thirties. The baseline interview questions did not change as the interviews progressed, though the interviewer probed around particular issues that were raised in previous interviews, such as frustrations with the health system. Once an issue emerged as being important in an interview (such as feeling frustrated with the healthcare system), it was probed for in all subsequent interviews. Before each interview began, interviewees were given information about the project and what taking part in it would practically involve (the approximate length of the interview and the types of questions that the interview would cover). Interviewees were informed that the interviews would be taperecorded (unless they had any objections to this), and that the interviews would be typed up and reported in an anonymous format. Respondents who completed face-to-face interviews were asked to give written consent to take part in the study. Respondents who completed telephone interviews were asked to give verbal consent.

The baseline questions were as follows:

•Can you tell me about your experience of managing your diabetes at the moment?

•How satisfied are you with your diabetes management at present?

•Do you have any concerns about your diabetes at present? If so, what are they?

•Do you have any concerns about the future? If so what are they?

•Can you describe your feelings about your diabetes at the moment?

•Does having diabetes, or having to manage it, ever upset you or get you down?

•Overall, what impact do you feel your diabetes has on your life at present? (negative or positive)

•What is the most difficult thing for you about having diabetes? What’s the best thing?

Interviews were thematically analyzed [41] in a word-processing package (MS Word). The first and the second authors coded the first four interviews and the other authors provided feedback on their analysis. Analysis for the first four interviews began by ‘open coding’ the interview transcripts, giving each section of a transcript that addressed a particular issue a descriptive tag or ‘code’ (e.g. a section of a transcript could be labelled ‘frustration with continuity of care’). These codes were compared and contrasted in order to determine if some of them could be subsumed under high level concepts or ‘categories’ (e.g. all posts labeled with the descriptive tags ‘frustration with continuity of care’ and ‘frustration with disjointed care’ were placed the higher level category ‘frustration with health services’). Eight principle categories were identified and agreed upon (outlined in Table 2). Information in subsequent interviews was evaluated against these eight categories. Although the first author was sensitive to the emergence of new major categories while analyzing the remaining thirty young adult interviews, it was determined that all codes from these interviews could be slotted under the initial eight categories. These eight categories became the organizing themes of the results section for young adults.

We checked the final draft of the article with the young adults who took part in the study. Young adults felt that the paper accurately described their experiences.

I’ve read over it is very interesting and very true (Female, 26).

Had a quick read through and it seems excellent, I certainly wouldn’t have any discrepancies (Female, 27).

## Results

### Sources of distress

Most interviewees considered diabetes to be emotionally difficult at least some of the time.

I’d be light hearted about it but at the same time, I’d be like sitting there going I wish I didn’t have this bloody thing (Male, 30).

### Self-consciousness about diabetes

A minority of young adults (n = 12) described feeling self-conscious about their diabetes and its management, and were worried about how others viewed them. The strength of these interviewees’ self-consciousness varied. Some felt ‘awkward’ when they had to manage their diabetes around others. Others had stronger feelings and perceived diabetes to be a stigmatizing, discrediting condition that risked undermining their identities as young, healthy people. These interviewees felt that their diabetes risked making them inalienably ‘different’ from other people.

A lot of people might feel a bit awkward if I was to inject in front of them (Male, 30)

People can see it, I think that’s the worst thing (Female, 27).

I don’t want to have to inject in front of them [strangers]. I think people do still find it a bit strange. It’s not heroin but it’s still a bit odd (Female, 23).

Interviewees who appeared to have strong stigma-related perceptions tended to avoid activities that they felt would highlight or reveal their diabetes to others. Such activities included wearing CSII devices (Continuous Subcutaneous Insulin Infusion), or, in the case of two young adults recruited from the clinical setting, joining diabetes-related support groups on social media sites such as Facebook. Avoiding such ‘stigmatizing activities’ enabled these interviewees to suppress their condition and allow them to present identities as ‘normal’ young adults in front of other individuals.

All my friends are on Facebook and I don’t want them to see that I have diabetes. Other people are probably like, ‘I don’t care if people see I have diabetes’. That’s why I wouldn’t join those groups [support groups]. With me, half the people I’m liking on Facebook wouldn’t have a clue that I have it (Female, 24).

Interviewees reported that their feelings of self-conscious were generally strongest during the first phase of young adult adulthood (18–22 years of age), becoming attenuated as they transitioned through their twenties. However self-consciousnesses could come to the forefront again during major life or environmental transitions, such as beginning a new job or moving to a new country. These were generally situations where interviewees either lost access to previously supportive audiences who could help to (re)frame diabetes management as a ‘normal’ activity, or forced interviewees to manage their diabetes in front of new and (potentially) unsupportive audiences.

It is kind of hard with working. You don’t want to be taking out a diary and writing down and people looking at you. I feel a bit uncomfortable. I don’t know the people that well inside there. In a new work environment I’d feel a bit uncomfortable. In my old work I would have cared less. I would have gone off and sat down for as long as I needed (Female, 23).

### Type 2 diabetes

Just under half of interviewees (n = 15) described feeling angry and frustrated at Type 2 diabetes. There were two reasons for this anger. The first was that interviewees felt that there a strong risk that they themselves could be misidentified as having Type 2 diabetes. Interviewees felt that the general public had a very prototypical and negative view of diabetes (derived from media reports of Type 2 diabetes that linked the condition to moral laxity, fatness, laziness, eating too much candy etc.). that they mapped onto all people with diabetes, including people with Type 1. These participants felt that they constantly had to differentiate between the two conditions to ensure that they were not stigmatized for having Type 2 diabetes. These interviewees themselves appeared to have negative perspectives of Type 2 diabetes; they seemed to see themselves as risk-avoidant, responsible subjects who developed diabetes through no fault of their own, whereas they thought that people with Type 2 developed their diabetes as a result of moral failings (i.e. inabilities to control their bodies and appetites). Notably, there were a number of interviewees (see Table 2) who were concerned about being stigmatized for having Type 2 diabetes but who did *not* feel self-conscious per se about having Type 1 diabetes (e.g. they had little concerns about injecting in front of other people as long as they were not mistaken for having Type 2 diabetes). It was also evident that whereas self-consciousness about Type 1 diabetes often attenuated as interviewees transitioned through their twenties, concerns about Type 2 diabetes appeared to remain strong.

That’s something that drives me crazy, Type 1 and 2 diabetes. It makes me so annoyed. Type 1 diabetes, you don’t get it because you’re overweight. People are like, ‘oh which kind do you have? Do you have the really bad kind?’ I’m like, ‘what do you mean by that?’ Or they’re like, ‘oh I’m sorry, did you eat too many sweets?’ Of *course* that’s why I have it (Female, 23).

We didn’t bring diabetes on ourselves. We didn’t have a choice. The majority of Type 2 s have a choice and they chose not to do what they should be doing. You feel like you’re branded with the same brush as them (Female, 30).

The second source of interviewees frustration was a feeling that Type 2 diabetes was receiving disproportionate attention and resources from the media, policy makers and charities. In contrast they felt that Type 1 diabetes was often neglected by these agencies and actors.

I really do think that Type 1 gets left behind. Even yesterday was world diabetes day and on the news it was all oh ‘if you improve your lifestyle and if you do this and that’ you improve your risk and I’m there shouting ‘this is Type 2 diabetes’! (Female, 28).

I think with diabetes a lot of it [money/resources] goes to Type 2 prevention, whereas Type 1 tends to get left in the dark and doesn’t get the services it should get (Female, 26).

### Day-to-day management

Day-to-day management of diabetes was considered to be emotionally difficult by just over half of interviewees (n = 18). Diabetes management frequently took up a lot of their time and energy, to the extent that other aspects of their lives were often neglected. Diabetes management had a visible component that other people could easily see, related to diet and injections. Underneath that, however, there was a larger element that other people were unaware of, the hidden, continually needed calculations and restrictions.

I don’t think people really understand what’s involved. It’s pretty much on my mind the whole time. There’s no day off from it (Female, 30).

I wouldn’t wish it on anybody. It’s awful. You have to do this and you have to do that. There’s more involved than just doing the finger when you eat (Female, 28).

It’s a huge thing. But no one can see it so it’s always there and it’s massive for you but it’s invisible for everybody else (Male, 24).

A majority of young adults (n = 21) considered diabetes to be a limiting influence on their lives, either preventing them from engaging fully in day-to-day activities such as work or forcing them to spend an excessive amount of time on diabetes management. Interviewees said that they often struggled to find an acceptable balance between their diabetes and their daily activities of their ‘normal’ lives.

I hadn’t drank for years because I didn’t know how with the diabetes. I didn’t go to festivals with my friends because I didn’t know what would happen with my diabetes there (Female, 24).

You feel like you’re dedicating a lot of your time and effort and emotions into this aspect of your life and other aspects are kind of being neglected. Stuff like college work can get affected and going out with your friends. You’re concentrating too much on this element and the rest of yourself is neglected a bit (Female, 27).

A number of female interviewees felt that they put on weight easily, more easily than their female friends and colleagues, which they attributed to their need to take insulin. They also noted that they had greater difficulties losing excess weight once they had acquired it.

I do find your weight does go up. I know it’s a side effect of insulin but since I’ve been on the pump, definitely my weight’s increased (Female, 28).

Interviewees who had difficulties controlling their diabetes were often particularly frustrated and upset: difficult control made interviewees feel anxious about the future; hypo-/hyper-glycaemic states could negatively impact mood; and it made interviewees feel that they were contravening norms governing how diabetes was ‘supposed’ to be managed. Poor control therefore could have primary (biological) and secondary (psychological) impacts on mood.

I just get fed up. Fed up injecting, fed up being tired. Sometimes I just feel like I’ve no life because I’m just exhausted. It’s not fair on the kids. It’s so hard to get up in the mornings because I have such high sugar readings (Female, 29).

No one understood how frustrating it can be sometimes when you’re doing your damndest to get it right and it’s not working. Because it’s your health on the line, it can be very upsetting (Female, 30).

Interviewees tended to blame themselves whenever anything went wrong with their diabetes and guilt appeared to be an ingrained feature of many interviewees’ diabetes management routines. Interviewees often seemed to see themselves as solely responsible for their diabetes management and felt that they had therefore personally failed when its management became suboptimal

I felt guilty because I knew I should have been stricter than I was. It was my own fault for my levels being high (Female, 27)

I’m always high or low. My sugars are never normal. I’m feeling crap then as well. That’s my own fault for not doing it. It’s just a big circle (Female, 24).

### Healthcare system struggles

A number of interviewees (n = 16) described feeling frustrated as a result of struggles with the healthcare system. Some interviewees described long fights with the health system bureaucracy to obtain diabetes technologies such as CSII. Others were frustrated by a lack of integrated healthcare services, forcing them to go to clinics on multiple occasions for different tests. Still others were angry at long waiting times between appointments. A common source of frustration was lack of continuity of care with doctors, and a lack of time with healthcare professionals (especially doctors) when they finally got to see them.

When I come away from clinic, I would never come away happy. You just feel so frustrated by the whole service and let down (Female, 28).

### Complications and the future

Over half of interviewees (n = 22) were concerned about what would happen to them in the future.

I have printed pictures of somebody who’s had their leg amputated up on my fridge. Any time I feel like having something bad I look at that and say, that will be you in twenty years time if you don’t keep yourself in check (Female, 29).

These interviewees had strong fears about developing diabetes-related complications, and lesser though still present fears about dying. Fears relating to amputation were particularly intense. Interviewees felt that their concerns about the future increased as they transitioned through their twenties: they realized that they were not invincible, and they became increasingly conscious about the possible consequences of any poor control that they had in the past. Many young adults in this study had also transitioned out of specialist young adult clinics and in to general adult health services; as such they were beginning to encounter- for example in hospital waiting rooms- older people with diabetes who had experienced amputation, blindness or other serious consequences of poor diabetes control.

That’s the fear, the complications of diabetes are awful (Male, 28).

I suppose because there were so many years that it was uncontrolled that I do kind of think, in the future will this effect kind of things when I’m older. Will I be one of those people that has to have their foot amputated (Female, 24).

I’d be sitting there beside an old man with a leg amputated and another blind woman beside you. It was so depressing. What the hell like? (Female, 29).

The unfairness of diabetes-related risk was of concern to some (n = 7) interviewees. These interviewees reported feeling anxious because there was only a probability, not a certainty, that things would work out for them and they would not develop diabetes-related complications if they took good control of their diabetes. Several of these interviewees made reference to other young adults with diabetes who they considered to be excessively risky (for example, taking drugs or binge drinking) and yet who did not appear to experience diabetes-related complications. It appeared that for these interviewees, diabetes was a universe where good deeds often went unrewarded and bad deeds unpunished.

And then there’s the whole long-term complications thing. I know you should look after yourself, but there’s no guarantees. There are sometimes when I do get stressed out about it… The friend that I mentioned, she does everything wrong and has had zero repercussions from it. She has no complications or anything. It just seemed a bit unfair (Female, 30).

I have necrobiosys in my arm and I think whenever my sugars are high I can see that worsening. It seemed a bit unfair. I was trying to do everything right. It just seems very unfair. I’d always try to take care of my sugars and do a bit better (Female, 30).

### Negative media representations

Media representations of Type 1 diabetes were a source of anger to a small number of interviewees. These interviewees felt the media representations of Type 1 were invariably predicated upon tragic models of illness, usually featuring older people struggling to live with the consequences of complications. Interviewees were quite angry about the absence of positive (or even balanced) media representations of Type 1 diabetes.

People like, articles and news reports tend to go with worse case scenarios and it’s scary. There are sometimes when I do get stressed out about it. There was one programme and they had some poor guy and he lost half a foot. I was like ‘Jesus Christ’. Then there was some poor woman and while they were filming it she died. I was like ‘holy crap’. It was very extreme. I don’t want to be told that by the time I get to 60 I’m going to have one leg and be blind. (Female, 30).

### Pregnancy

Pregnancy-related concerns were common amongst female interviewees. Interviewees were particularly concerned about miscarrying and the risk of having large babies, and the effects that this largeness could have on their own health and on their baby’s. Interviewees were also afraid that they would become pregnant during a period where they had poor control.

The whole idea of having a baby was going to be a new experience and really exciting and now for me it’s really dampened by the fact that I’m going to be even more conscious of my diabetes (Female, 30).

What’s the point in trying when I could have a miscarriage. I think its 60/40 that I’ll have a miscarriage more times than I’d actually have a baby. It does my head in basically (Female, 24).

### Managing distress

Interviewees used a number of strategies to manage diabetes-related distress. One was to avoid- as far as possible- thinking about the negative aspects of diabetes. A reason why interviewees appeared to became so annoyed at negative media representations of diabetes because they disrupted their attempts to focus on the positives.

I think a lot of time so much is focused on the negatives and I don’t really like to think about that (Female, 23).

I do think it would be good to look at the positive, success stories, if there are any (Female, 30).

Interviewees also made downward comparisons with people who they considered to be less fortunate than themselves, which served to represent their own circumstances in a more positive light. Some interviewees compared their current diabetes management practices with their past practices in order to demonstrate that their diabetes management had improved with time and they were on an upwards rather than downwards trajectory (this helped to manage fears about the future and developing complications).

Well there’s no real point in moping around, ‘oh God, poor me’. Just get on with it. There’s people way worse off. There are way worse diseases than diabetes (Male, 30)

For years I didn’t test at all so I’m like, at least I’m testing. To me it’s ok. It could be better but it’s ok (Female, 30).

Another strategy was to put a sustained, conscious effort into seeking to develop increased control over diabetes, for example through acquiring diabetes technologies such as CSIIs or seeking to go on structured diabetes education courses. Knowledge acquired from structured courses seemed to help to empower interviewees, helped them to feel that diabetes management problems were errors that could be fixed rather than the outcomes of personal failings, which in turn ameliorated feelings of guilt and frustration.

I’m a lot more relaxed about it than I had been. Like I was constantly frustrated about it, why isn’t it working. But now [since the structured education course], if it’s high, before I would have gotten really annoyed and sometimes even upset if my sugars were high but now I go, ok obviously I miscalculated how much that piece of cake was and then I learn from that (Female, 30)

### Social support from healthcare professionals

One strategy for managing diabetes-related distress was present in most young adults’ accounts, and therefore we will consider it in more detail here. That solution was to obtain diabetes-related social support, which young adults received from three main sources: healthcare professionals, family members and peers with diabetes.

Young adults felt that having opportunities to talk to doctors and nurses often helped them to manage their feelings of diabetes-related distress.

I think the nurses don’t get enough credit. There’s the social aspect [to what they do], the caring side (Female, 23).

Young adults also thought that healthcare professionals should provide emotional support to young adults as part of their standard care.

I honestly think there should be some kind of emotional support. That’s part of your health that should be addressed in the clinic (Female, 23).

However many interviewees noted that they rarely had the time or opportunity to discuss their diabetes-related emotional problems in clinic appointments. Appointments with doctors were often brief, occurred only once or twice a year, and young adults often saw a different doctor every time that they went to their diabetes clinic. Such doctors were often junior doctors in training who lacked expertise in diabetes and the psychosocial issues associated with the condition. Many young adults said they were uncomfortable bringing up diabetes-related distress problems with doctors who were effectively strangers. Appointments with doctors often focused on processing biomedical information, such as Hba1C scores, with emotional and psychosocial issues bracketed to one side. Appointments with doctors who young adults had not met before were often taken up with going over historical information, such as date of diagnosis, which further reduced the time that doctors had to explore young adults’ feelings.

You go in and see the registrar and they don’t know who you are. You don’t feel open or comfortable talking to him about how you’re getting on. I think seeing the same person helps big time (Female, 23).

When you go to the doctor they’re all about the diabetes, looking after your control there’s nobody there to talk to say ‘have you had enough of it?’ That would be something that would really help. A bit of support for that would be really worthwhile. To know that it’s not only you (Female, 29).

There was one or two doctors that made flying visits in and out and didn’t really treat you like a patient. It was more like you’re a file. They just have a quick look at the file and look at numbers without discussing any of the issues with you (Female, 27).

Young adults’ relationships with nurses were often better, but again the support that nurses provided was often not enough, only occurring in the context of brief clinical encounters that took place every six months to a year.

I mean my diabetic nurse got me through it all. She was amazing, absolutely amazing (Female, 28).

The nurse specialists were brilliant. (Female, 30).

### Support from family members

Young adults also received diabetes-related social support from their families. The support provided by families was considered to be important by most interviewees. Families provided *tangible support,* mainly in the form of taking note of interviewees’ health, particularly their blood sugar numbers and what they were eating. Tangible support was important because it helped to increase interviewees’ feelings of mastery over their diabetes, and also helped reduce anxieties relating to acute diabetes complications such as hypoglycaemia.

My wife would be what you’d call a concerned wife. She would be very strict around diet so she’s good at keeping me in line and monitoring what I’m eating (Male, 30).

Family members also provided young adults with emotional support. Emotional support manifested itself in several ways. Family members often reassured young adults that everything would ‘work out o.k.’ for them, which helped interviewees to regulate any feelings of anxiety that young adults had about developing diabetes complications. Family members also actively intervened to protect young adults from distressing items in their environment, such as negative media representations.

I went to this diabetes focus group, there was two people sitting there and they were both talking about having complications. I came home and was like to mum, ‘oh God, I’m nowhere near that stage in my life but still that’s kind of a scary thought to think that could have complications’. But then, as my mum said, loads of people who don’t have any other illness have complications or can’t get pregnant (Female, 23).

My mum was recording this programme and something came up on diabetes and complications. She deleted it because she knew if I saw it I’d have a fit (Female, 24).

However family support on its own was considered to be insufficient by many interviewees. The interviewees who took part in this study were in the second phase of young adulthood and had often left their family homes, which meant that they usually received less intensive support from their families compared to when they were younger. Family members sometimes themselves became very anxious about young adults’ diabetes management, forcing young adults to spend as much time regulating their family members’ emotions as they did their own. Young adults felt that family members’ support could at times edge towards social control attempts. Family members were also often felt to lack an understanding of what it was like to manage diabetes; family members’ supportive attempts therefore sometimes miscarried as they did not consider the impact that their actions would have on young adults.

I think my family don’t really understand the mental side (Female, 27).

I went on holidays in August and my aunt texted me to say there was an article in the Irish Times to say diabetics shouldn’t wear sandals because of the risk to their feet. I was like, have a bit of common sense. I was so annoyed over that. I was like, have a bit of tact (Female, 30)

I just get anxious and over think things. At the weekend it would be like, I kind of spend most of the day in bed. Just because I skip meals and then sometimes I just go out and buy junk and stay in my room. I could go home to my family but then I end up getting stressed out down there. Just anxiety and stress (Female, 25).

### Support from peers

As was reported in the methods section, a large number of interviewees joined diabetes support groups on the Internet, especially on Facebook. Interviewees felt that other people with diabetes had empathy for their experiences and would not judge them or become anxious if they admitted that they had problems. Most interviewees said that they wanted to receive support from people who were their own age and who had Type 1 diabetes; they did not want to become involved with older people with Type 2 diabetes, either in terms of giving or receiving support.

It’s great to have someone who understands (Female, 30).

I think the best thing ever is to have someone else who’s in the same boat as you (Female, 30).

Peers with diabetes helped young adults to regulate negative emotions. They provided young adults with positive examples of living with diabetes, examples that highlighted that living with diabetes was often difficult, and that interviewees were not alone in their suffering. Peers also provided young adults with practical information and motivation that interviewees could use to improve their diabetes control, which in turn could help them to reduce their feelings of being overwhelmed by their diabetes.

X kept me sane, she was like, you’re not doing badly, calm down. It’s one thing to hear it from doctors but when it’s somebody else who’s actually been through it (Female, 30)

It’s supportive to hear other people say that they’re not all perfect at managing their diabetes. It’s hard. (Female, 28).

Another advantage of peer support was that it was much more readily and easily attainable than support from healthcare professionals, who as noted previously often only saw interviewees several times a year. In contrast, some interviewees were able to receive peer support several times a day, whether from online or offline sources.

I’ve spoken to three or four people through the Facebook group who’ve just been recently diagnosed and they’ve had questions for me. I’ve said ring me any time, I don’t mind if you need to talk to someone and that alone is a relief to hear, to know that there’s people there to talk to, again, because no one else really gets it (Female, 30).

There were two notable issues about diabetes peer-support groups, however. One was that they were composed of untrained individuals who were sometimes quite young and quite distressed about their diabetes. One female interviewee, who helped to run an Internet support group, felt imprisoned by a need to convey a positive impression to other young adults with diabetes. So while she joined peer groups to receive emotional support, she ended up becoming more distressed out of a need to demonstrate to other young adults that diabetes was not distressing.

I’m not a support worker. I’m quite worried that I’m not providing enough support for these people. I find it difficult to approach someone who I can see is having a problem. I think they [other young adults with diabetes] expect me to have it perfect. I don’t know. People have said when they meet me, it’s great, it just shows that diabetes doesn’t have to wear you down. I don’t really want to admit to them if I’m struggling. I feel like I play this role, they look at me and go, there’s hope there so I don’t want to ruin that for them (Female, 27).

Secondly, a number of young adults noted that peer-support groups required resources for them to be optimally successful. At a minimum, real-life support groups required rooms in clean, well-lighted places where meetings could be held. Support groups- both online and off-line- also demanded significant amounts of time and effort from moderators and group organizers. These time demands could at times cause group moderators to experience some difficulties, as they usually ran these groups while working full-time jobs and juggling other commitments such as children. Several peer-group organizers who we interviewed noted that they had approached hospitals to ask for small amounts of funding to support their groups; however while hospitals were often enthusiastic about the idea of young adult support groups, they said that they were unable to fund them in any way.

I said to a consultant, ‘I know the Irish health system won’t want to know because it may cost money’. He just laughed and was like, forget it. He said he can’t get what he needs and he is the hospital, not a support group (Female, 30).

### Distress is not an issue

Finally, four interviewees (see Table 2) indicated in their interviews that they were not distressed either by having or by managing diabetes.

I’m not stressed about it [diabetes] (Female, 30).

I was fairly laid back about it [diabetes] (Male, 30).

I’ve had it long enough that it seems like I’ve always had it. I don’t remember it ever being hugely problematic (Female, 25).

Two of these interviewees were male, two were female. Three were thirty years of age, one twenty-five. Three were married, the other in a long-term relationship. Three had been diagnosed with diabetes in their twenties, the other in her late teens. All indicated that they had positive relationships with their healthcare professionals. Two worked in finance; the other two had worked in finance in the past, and at the time that the study was conducted had gone back to University (both to study science subjects). All four therefore appeared to have support from the official health system, had stable personal lives, were educated, had quantitative backgrounds. All four appeared to take active problem-solving approaches to their diabetes, with three of the four noting that they preferred to solve diabetes-related problems for themselves rather than contact their diabetes clinic.

I’ve a maths degree originally, so I was working out tables for how much sugar to set off against the carb intake and working out how much carbs I was eating and so on (Male, 30).

I would be the kind of person who’d just figure it out myself. But you know, sat down, read what the disease was, how it’s generally treated and how the regimes work (Male, 30).

These interviewees also described themselves as being flexible and able to deviate from diabetes management guidelines without becoming anxious. They noted that they had adjusted very quickly to having diabetes. Two of these interviewees felt that that their lifestyles changed relatively little after they were diagnosed, and one said that his brother had previously been diagnosed with diabetes, which meant that his life had already been preadapted to diabetes to some extent prior to diagnosis. These interviewees also felt that they were psychologically resilient and able to deal with adversity without becoming overwhelmed.

It wasn’t such a devastating thing at the end of the day. That’s my personal opinion on it (Male, 30).

I think I’ve always been quite, most people would say, too laid back a person. I always manage my stress levels very well. I suppose I just try and take it all in my stride and really not stress about it (Male, 30).

## Discussion

This study helps to address the lack of research on the causes of diabetes-related distress in young adults in their 20s. Quantitative research suggests that diabetes distress is an important issue in this age group [4,7]. The utility of the study’s findings are that they provide a list of topics that healthcare professionals can use to start dialogues with these young people.

A range of sources of distress were identified by this project, including: stigma/self-consciousness; concerns about Type 2 diabetes; day-to-day management difficulties; struggles with the healthcare system; fears about the future; negative media portrayals of diabetes; and concerns about pregnancy.

Previous research has identified several of these factors as being distressing to people with Type 1 diabetes, though not necessarily to young adults in their twenties. People with diabetes have been noted to become distressed when they feel restricted or disadvantaged [42]. Many find the unremitting nature of day-to-day management to be emotionally difficult [32]. A number of young adults feel awkward managing their diabetes around other people and a minority appear to fear that other people will not accept them [1,42,43]. As noted, some interviewees in this study were either concerned that they would be stigmatized for having Type 1 diabetes, or concerned that they would be mistakenly stigmatized for having Type 2 diabetes. Several young adults themselves appeared to stigmatize other people with Type 2 diabetes. Schabert et al. [44] note that diabetes-related stigma-whatever its source- can have significant negative impacts on people with diabetes’ psychological well-being. The findings here suggest that stigma is not only a serious problem itself, it can also discourage young adults from seeking help for diabetes-related distress; one female interviewee, for example, noted that she would not join Facebook support groups in order to protect her identity.

Young adults’ concerns about being mistaken for having Type 2 diabetes likely reflect wider negative social understandings of Type 2 diabetes as a ‘ticking time-bomb’, and people who develop Type 2 diabetes as risky, uncontrolled and obese subjects. It was evident that interviewees were anxious that the general public would be unable to differentiate between Type 1 and Type 2 diabetes. Interviewees’ anxieties here could have been further accentuated by the fact that many young women with Type 1 diabetes appear to experience increases in their body mass index as they transition through their twenties [45]. These young women may therefore feel that if other people see someone with a ‘large’ body, and associate that body with the word ‘diabetes’, they will assume that the young person has developed diabetes as a result of an inability to self-regulate. It was notable as well that some young people felt that people with Type 1 diabetes were competing for resources with people with Type 2 diabetes. Competition for scarce economic resources often intensifies stigmatization processes and may have contributed to some interviewees’ hostility towards people with Type 2 diabetes [46].

Fears about the future have previously been found to hang over the heads of some young people with diabetes like a ‘sword of Damocles’ [1,23,32]. Complications can be distressing not only in and of themselves, but also because they can undermine young adults’ life plans and career aspirations [43]. Young adults have also been noted to be concerned about the complicating impacts of diabetes on pregnancy [1,28]. Other studies have indicated that young adults can become very distressed at hypoglycaemia, though this was something that we did not detect to any great extent in this study [1]. The young adults who we interviewed reported being much more anxious about developing hyperglycaemia than they were about ‘going low’. Young adults’ developmental position may help to explain some of these findings. As noted, the young adults in this study were in the second, ‘stabilizing’ phase of young adulthood. Previous theoretical research suggests that young people in this age range become increasingly concerned about their diabetes management [3,28]. They tend to lose their adolescent sense of invulnerability. Simultaneously, they are transitioning from young adult to adult health services where they are either sometimes beginning to develop diabetes-related complications themselves, or beginning to see the consequences of such complications in other people. They desire to build positive relationships with healthcare providers and engage with their healthcare system. This theoretical work would suggest that fears about the future become increasingly salient for individuals in this age group, more than anxieties about short-term complications, as would fears about pregnancy (young adults in their twenties would be at an age where they would seriously be beginning to consider having children, with all of the risks and complications that entails). It would also suggest that young adults- who wish to engage with health services in order to improve their control and to reduce their risk of developing complications- would become distressed if they perceive services to be unresponsive to their needs.

Overall, the findings of this study indicate that individuals in the second phase of young adulthood can experience significant diabetes-related emotional struggles. However, clinicians have often not paid the same attention to the emotional problems of these individuals as they have to their younger counterparts, those in their late teens and early 20s. ‘Older’ young adults in their mid to late 20s are often treated in adult services where psychological support is absent, where continuity of care is lacking and where interactions with professionals are focused on HbA1c scores and other clinical measures [4]. It was notable in this study that although participants experienced diabetes-related distress, they also generally had reasonably good diabetes control. Although we cannot state this point definitively (as the study is based exclusively on patient perceptions of healthcare professionals’ practices), it may be that some clinicians and other professionals are briefly interacting with these young adults, seeing reasonably good HbA1c scores, thinking that everything is acceptable, and moving on to the next patient; or are aware of distress in this group but do not feel that that they have the time or the expertise to deal with these issues within the context of regular clinical appointments [11]. It may therefore be that distress in more ‘successful’ young adults such as the ones in this study is either being elided or bracketed by some professionals.

It is important that healthcare professionals address diabetes-related distress in young adults with Type 1 diabetes, particularly given that distress is associated with poor clinical outcomes in many patients [2] – though there are subsets of patients for whom distress may have beneficial impacts upon self-care, possibly by encouraging young adults to regulate themselves in order to reduce feelings of anxiety [4,46]. Gonzalez et al. [2] suggest that the best way for professionals to manage diabetes distress may simply be to have brief, direct and ongoing conversations with patients. Finding the time to do so may be difficult in the context of a typical clinical appointment; nevertheless it is important. The findings of this study also suggest that it could be important to offer young adults opportunities to attend structured diabetes education programmes [47] and provide them with access to technologies that they can use to improve their control such as CSII. All of these suggestions come with cost and resource implications. A number of researchers have indicated that an efficient way of helping young adults to manage distress may be by helping them to develop and maintain peer support networks [32,48]. However the findings of this study indicate that volunteer-led peer support groups should not be seen as a way to provide young adults with ‘free’ social support. These groups require resources and inputs from volunteers, even if it is just in terms of their time and effort. It is unclear how resilient these peer-support networks will be over time without support (money/training/encouragement) from the official health system. The young adults who run them will grow older, and possibly less willing to supply peer-support to younger adults who are experiencing issues that they have ‘grown out’ of. Finally, the findings of this study suggest that it may be useful for clinicians and diabetes researchers to engage with the media to ensure that representations of diabetes are not overwhelmingly negative, and that Type 1 and Type 2 diabetes are sufficiently differentiated.

The main limitation of the study is the self-selected nature of the young adult sample (as Table 1 highlights most of the sample was composed of educated young women with reasonably good control). Another limitation was that the sample was recruited from Facebook and may reflect a particularly engaged cohort of young people (those engaged with their diabetes to seek Internet support or information). Some of the solutions to diabetes distress that respondents proposed, such as peer support groups, may not be relevant for other individuals for whom interacting with peers with diabetes is not so important. We did not assess the intensity of young adults’ diabetes-related distress; some young adults who felt angry at Type 2 diabetes might have been very angry at Type 2, others ‘merely’ annoyed. It is difficult to definitively state if, and if so how, the interviewer’s identity impacted the information that was disclosed in the interviews. However it may have been that some very sensitive issues, such as distress arising as a result of psychosexual issues or eating disorder behaviours, were not revealed to the male interviewer by the predominantly female group who took part in this project.

## Conclusions

Many aspects of managing diabetes distress young adults with Type 1 diabetes who are in their twenties. The most common factors identified in this study were stigma, day-to-day diabetes management difficulties, concerns about the future and apprehension about pregnancy. Young adults felt that diabetes distress was often missed during clinical appointments. A number of factors may help to moderate distress in young adults in their 20s, including having opportunities to talk to healthcare professionals, attending diabetes education programmes and joining peer support groups. Further research is needed to determine which interventions are most effective in addressing distress in this age group. It would also be useful if future research explores why some young adults do not become distressed as a result of living with a condition such as Type 1 diabetes.

## Competing interests

The authors declare that they have no competing interests. This study was funded by a grant from Diabetes Ireland, the MRCG and the HRB.

## Authors’ contributions

All authors were involved in the conceptualization and the design of the study. MB carried out the interviews. MB and FD analysed the interviews with SS, DS, RB, DH and RC commenting on their analysis. MB and FD drafted the manuscript and SS, DS, RB, DH and RC revised it. All authors read and approved the final manuscript.

## Pre-publication history

The pre-publication history for this paper can be accessed here:

http://www.biomedcentral.com/1472-6823/13/25/prepub
